# Ocular Manifestations of IBD: Pathophysiology, Epidemiology, and Iatrogenic Associations of Emerging Treatment Strategies

**DOI:** 10.3390/biomedicines12122856

**Published:** 2024-12-16

**Authors:** Holly Richardson, Giho Yoon, George Moussa, Aditi Kumar, Philip Harvey

**Affiliations:** 1 Department of Undergraduate Medical Education, The Royal Wolverhampton NHS Trust, Wolverhampton WV10 0QP, UK; 2 Manchester Royal Eye Hospital, Manchester M13 9WL, UK; george.moussa@mft.nhs.uk; 3 Department of Gastroenterology, The Royal Wolverhampton NHS Trust, Wolverhampton WV10 0QP, UK; aditikumar@nhs.net

**Keywords:** inflammatory bowel disease, Crohn’s disease, ulcerative colitis, extraintestinal manifestations, episcleritis, scleritis, uveitis, keratoconjunctivitis sicca, peripheral ulcerative keratitis

## Abstract

Inflammatory bowel disease (IBD) is a complex, multisystemic disease and is associated with ocular pathology in 4–12% of patients. In general, ocular disease affects Crohn’s patients more frequently than those with ulcerative colitis. Episcleritis and uveitis are the most common presentations, with episcleritis often correlating with IBD flares, whereas uveitis presents independently of IBD activity and, in some cases, may even alert clinicians to a new diagnosis of IBD. Corneal EIMs encompass a range of pathologies, such as the common and benign keratoconjunctivitis sicca (dry eye disease), which nevertheless causes significant patient discomfort, and the rarer condition of peripheral ulcerative keratitis, which warrants urgent review due to the risk of corneal perforation. Alongside EIMs, clinicians should also be aware of the iatrogenic consequences to the eye following treatment of IBD. Corticosteroids may cause cataracts, glaucoma, and—indirectly via hyperglycaemia—diabetic retinopathy. Methotrexate is irritating to ocular tissues and may cause conjunctivitis and blepharitis. Biologic medications, such as anti-TNFα agents, overlap in their use as treatment of both IBD and uveitis, and yet in some patients may also increase the risk of acute uveitis flares, as well as opportunistic, sight-threatening infections. With integrated care between gastroenterology and ophthalmology, patient outcomes can be improved by facilitating earlier detection and management of ocular disease. This narrative review summarises the ocular extraintestinal manifestations of IBD, including pathophysiology, epidemiology, and current treatment strategies.

## 1. Introduction

Inflammatory bowel disease (IBD) is a complex disease affecting the gastrointestinal tract with multisystemic effects. It is immune mediated, chronic, and follows a remitting-relapsing pattern [1]. These characteristics are shared by the two discrete conditions that IBD encompasses, ulcerative colitis (UC) and Crohn’s disease (CD).

Although IBD predominantly affects the gastrointestinal tract, its effects on other organ systems, known as extraintestinal manifestations (EIMs), carry significant morbidity. These include, but are not limited to, the musculoskeletal, dermatological, hepatobiliary, and ophthalmic systems [1,2]. The literature varies regarding the prevalence of EIMs [3,4,5,6]; however, it is likely that up to half of patients will be affected by an EIM in the 30 years following IBD diagnosis [7]. Ocular manifestations are the third most common EIM, following musculoskeletal and mucocutaneous pathologies [8].

**Figure 1:**
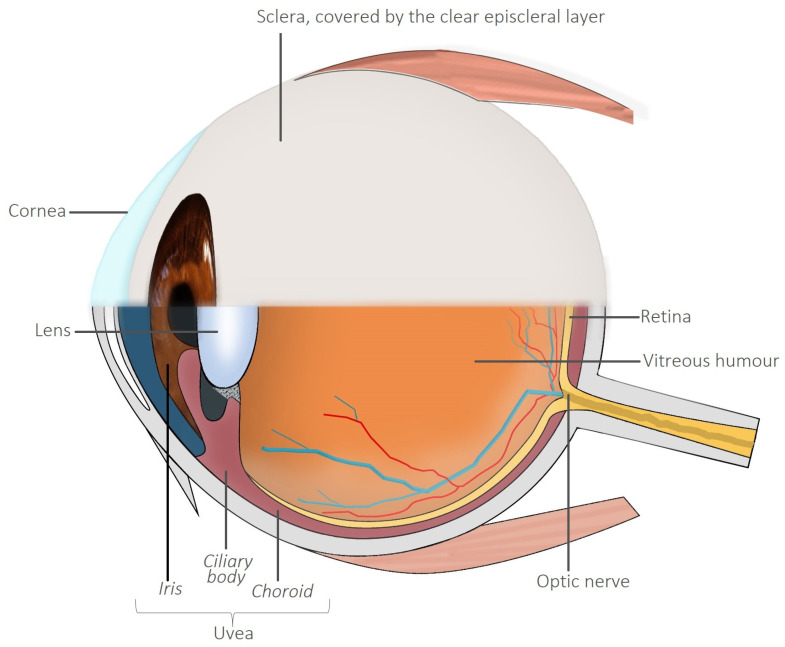
An illustration of important ocular anatomical structures. The uveal tract comprises the iris, ciliary body, and choroid. Original image.

Ocular manifestations are more common in patients with CD than UC, those with a longer disease duration, and smokers [9]. They typically affect 4–12% of patients [2]; however, in some studies, the prevalence is as high as 29% [10]. Although episcleritis and anterior uveitis are the most common [6], manifestations such as scleritis are also important to recognise due to the risk of damage to ocular structures [1,6] (Figure 1 shows the anatomic structures of the eye and their relationships). The temporal association of IBD and ocular EIMs is frequently unpredictable. Ocular disease may precede intestinal symptoms in some cases, and, therefore, may facilitate early diagnosis of IBD [1]. Iatrogenic eye disease is another important consideration, as corticosteroids, immunomodulators, and biologic therapies may cause unintended ocular sequelae.

This narrative review will provide an overview of the common ocular manifestations of IBD, including the pathophysiology, epidemiology, presentation, and current management options. It will additionally consider the emerging evidence on associations between new IBD treatment strategies and ocular disease.

## 2. Episcleritis

### 2.1. Overview

Episcleritis is an inflammatory condition of the episcleral vascular layer between the sclera and conjunctiva. It can be simple or nodular, but with up to 75% being simple, this is the most common subtype [11]. The pathophysiology of the condition is characterised as a non-granulomatous inflammation of the episcleral layer [12].

### 2.2. Epidemiology

Episcleritis is the most common ocular manifestation of IBD, typically seen in 2–5% of patients [13]. It often manifests during acute phases of IBD and, therefore, serves as a useful indicator of disease activity [12,14]. Globally, episcleritis is seen in equal frequency in CD and UC; however, in European populations, it is more than twice as prevalent in CD compared with UC (pooled OR 2.40, 95% CI 1.11–5.19, *p* = 0.026) [15]. Proposed explanations for this include greater inflammation in CD, reduced fibre intake, increased meat intake, and urbanisation [15,16].

Interestingly, within the demographics of race, age, and gender, there are no significant differences between episcleritis and the more severe scleritis [17]. Due to the similar patient risk factors and demographics that present with each condition, the distinction between the two must be made clinically.

### 2.3. Presentation

Simple episcleritis presents with symptoms of grittiness and occasionally photophobia, with sectoral or diffuse redness of the eye on examination. This condition is milder in symptomatology compared with other causes of red eye, such as scleritis, and typically does not affect visual acuity. Furthermore, up to half of patients with simple episcleritis have no ocular discomfort and at worst report moderate discomfort [11].

Nodular episcleritis is similar but is slower in onset and longer in duration. Patients report a characteristic painful red nodule, usually within the interpalpebral fissure [11].

Episcleritis can be mistaken for other conditions causing an acute red eye, such as conjunctivitis and scleritis. It is imperative to be able to differentiate between these conditions, as accurate recognition directly affects treatment and prevention of complications [12,17]. Episcleritis can be distinguished from scleritis by the application of 10% phenylephrine drops, which will blanche the dilated episcleral vessels, resulting in the sclera returning to a healthy white colour. This contrasts with scleritis in which the eye will remain hyperaemic and red [11].

### 2.4. Management

Both simple and nodular episcleritis are usually self-limiting, with patients advised to use cool compresses and topical lubrication for symptomatic relief as the conservative first-line therapy [1,11]. Reassuringly, patients will often see resolution of episcleritis with appropriate treatment of underlying IBD [14,18,19]. Topical or oral non-steroidal anti-inflammatory drugs (NSAIDs) and topical corticosteroids can be used in refractory cases, although NSAIDs may predispose to IBD exacerbations and adverse gastrointestinal effects and are not currently recommended by the European Crohn’s and Colitis Organisation (ECCO) [14,20,21]. In a case study, infliximab was shown to be effective in treating refractory episcleritis in a patient with CD [14].

## 3. Scleritis

### 3.1. Overview

Scleritis is an inflammatory disease of the sclera, which is the opaque white layer of the eye, and the underlying scleral vasculature [12,14]. The condition has historically been classified into anterior scleritis and posterior scleritis, with the former being further subdivided into diffuse, nodular, necrotising with inflammation, and necrotising without inflammation [22].

While the role of the immune system in the development of scleritis is well recognised, there is limited understanding as to its precise pathophysiology and relationship with other systemic autoimmune diseases [23]. Similarities in predisposing genes and autoimmunity due to overlap in the composition of organ tissue are proposed hypotheses that require further exploration [23].

### 3.2. Epidemiology

Scleritis is rarer than episcleritis, occurring in less than 1% of IBD patients [3,13], with older patients, female patients, and those of Black or South Asian ethnicity at higher risk [24,25,26]. Scleritis is associated with multiple immune-mediated inflammatory diseases, with scleritis patients in the UK over three times more likely to have a diagnosis of CD than a control group (OR 3.60 95% CI 2.28–5.67) and more than twice as likely to have UC than controls (OR 2.20 95% CI 1.49–3.27) [25].

### 3.3. Presentation

**Figure 2:**
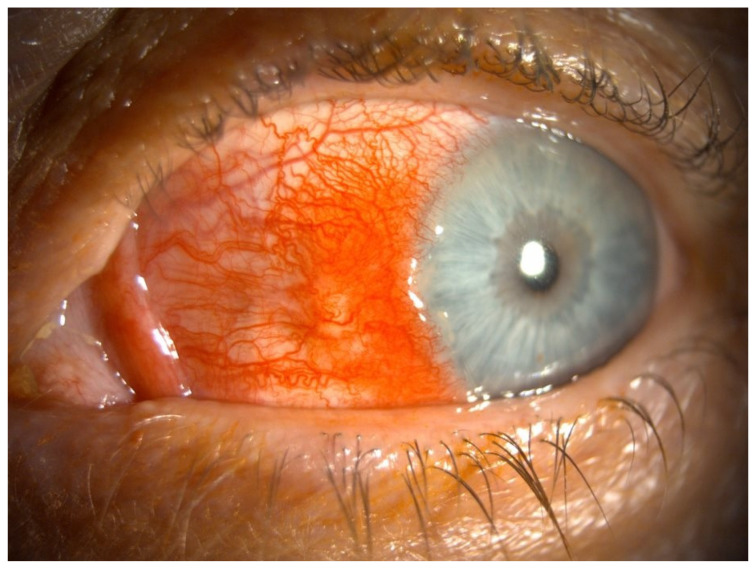
Episcleritis (pictured) and scleritis both present with dilation and increased visibility of the vascular congestion of the superficial episcleral plexus and deep vascular plexus. Application of phenylephrine drops would cause constriction of the superficial vessels involved in episcleritis but not the deeper vascular plexus as with scleritis. *Image kindly provided by the Manchester Royal Eye Infirmary*.

Scleritis presents with redness of the sclera which progresses to severe eye pain, commonly described as deep or throbbing in character, with blurred vision and ocular tenderness [11,12,22]. Compared to episcleritis, the symptoms are typically more severe, may wake patients from sleep, and improve throughout the day [11]. Examination reveals redness of the sclera, which remains unchanged with application of topical phenylephrine and displays a characteristic ‘blue hue’ with recurrent disease [1,20] (see Figure 2). A further diagnostic test is the manipulation of the inflamed vasculature with cotton tip applicators, which will be movable in episcleritis, but it is not possible to manipulate in scleritis due to the anchoring of the scleral vasculature to the globe [1]. The hyperaemia may be restricted to a specific area of the sclera or diffusely distributed across the eye. Scleral nodules may also be observed, usually in the interpalpebral region [11].

Clinicians should be vigilant of the challenges in recognising posterior scleritis, which can be investigated using ultrasound imaging to detect fluid in the subtenon space [11]. The non-distinct presentation, with inflammation occurring behind the examinable aspects of the sclera, can lead to difficulty in diagnosing this subcategory of scleritis [27,28]. Some cases may present with more significant signs such as ptosis, retinal detachment, diplopia due to restriction of extraocular muscles, or raised intraocular pressure [27]. Although posterior scleritis is a less common form of scleritis, the high risk of visual impairment suggests a low threshold is needed to investigate patients with atypical presentation, such as those with disproportional levels of pain or double or reduced vision [17,29]. Younger patients, female patients, and those with a history of anterior scleritis are more predisposed to posterior scleritis [30,31].

### 3.4. Management

Scleritis, when associated with systemic disease, is more likely to necessitate extensive and aggressive management [32], while also being more likely to lead to visual impairment [17]. This is particularly pertinent, as up to 50% of patients with scleritis have an associated systemic disorder [17]. An IBD patient with suspected scleritis (for example, waking from sleep due to eye pain) thus warrants urgent referral to an ophthalmologist [14,21]. Scleritis should be treated aggressively with oral NSAIDs (first line) and oral corticosteroids (second line) [13,14,21,32]. Immunosuppressants and biologics are considered in refractory cases [21,32]. In a retrospective study, nearly 90% of patients required systemic therapy (NSAIDs, prednisolone, or systemic immunosuppressants), with around 25% of patients requiring immunosuppressants [17]. This study was undertaken in a tertiary centre and, therefore, included more complex cases; yet, it highlights the frequent inevitability of systemic scleritis treatment.

Topical therapies such as topical NSAIDs and topical corticosteroids, are also occasionally used [1,21]; however, evidence is weak on the effectiveness of topical NSAIDs [33]. In contrast, topical corticosteroids have demonstrated effectiveness in symptom improvement as an adjunct to systemic therapies but should be used with caution due to the risk of developing cataracts and ocular hypertension [1,33].

In aggressive cases, for example, necrotising scleritis, in chronic disease, or in cases refractory to NSAIDs and corticosteroids, immunotherapy may be used to improve visual prognosis [11,32]. However, the evidence base for selecting immunotherapy regimes is not robust [32]. Methotrexate is considered an appropriate starting point, or azathioprine or mycophenolate mofetil may be alternatively used if there are significant side effects or refractory cases [18,32]. These suggestions are echoed by the ECCO consensus [18].

The use of biologics can also be beneficial in the treatment of scleritis. Infliximab is effective and may improve visual acuity or inflammation in over 90% of cases, with 64% of patients able to reduce or discontinue corticosteroid treatment and 36% able to discontinue infliximab entirely [32]. Ultimately, when associated with a systemic disease, such as IBD, it is important to ensure adequate treatment of the underlying condition to ensure the chance of recurrence is minimised [33].

Scleritis is not always present during periods of active intestinal flares and can occasionally precede systemic disease [20,34]. Due to its association with other systemic inflammatory diseases, such as rheumatoid arthritis and vasculitis, it is important to ensure a thorough examination and appropriate speciality referrals wherever necessary [20,24]. The complications of recurrent scleritis are significant, as it may eventually cause ocular perforation [1]; therefore, early recognition, treatment, and control of underlying autoimmune systemic conditions, including IBD, is imperative in the long-term management of this condition [20].

## 4. Uveitis

### 4.1. Overview

Uveitis is a group of inflammatory diseases affecting the middle layer of vascular structures of the eye. It is the most common ocular EIM and frequently precedes IBD diagnosis [35]. Importantly, uveitis is independent of the activity of the intestinal disease [7,12].

Uveitis may be categorised according to aetiology. Around 30–40% of cases are non-infective and associated with autoimmune inflammatory diseases, such as IBD, psoriatic arthritis, or Behçet disease. Infection, including bacterial, viral, and fungal, and trauma is a separate but significant cause [36,37]. Uveitis may be further subdivided according to the structures that are affected. Anterior uveitis is the most commonly associated with IBD and involves inflammation of the iris (iritis) and sometimes also with involvement of the ciliary body (iridocyclitis). Rarer forms of the disease include intermediate uveitis, which affects the vitreous humour, and posterior uveitis (chorioretinitis), which is inflammation of the retina and/or the underlying choroid—the network of blood vessels supplying the retina [12]. A combination of diseases may be referred to as panuveitis [1].

The pathophysiology of uveitis is complex and multi-factorial. Studies suggest that multiple genetic factors are involved and likely triggered by a pro-inflammatory gut microbiome, including *Prevotella* and *Streptococcus* species [12]. These gut flora are thought to trigger the activation of autoreactive T cells, which have a causal link with uveitis [38]. Cytokines, including interleukins IL-6, IL-10, and IL-17, play a role in the pathophysiology of both uveitis and IBD, suggesting that these may circulate to the eye and cause uveal inflammation [39]. Additionally, a reduction in *Clostridioides* and *Bacteroides* in patients with IBD can lead to a decrease in de-hydroxylated bile acids, which are suggested to have an anti-inflammatory effect in preventing uveitis [40]. A disrupted microbiome can similarly lead to decreased production in the gut of short-chain fatty acids (SCFAs), which are associated with lower rates of uveitis [12].

Despite anti-inflammatory properties, anti-TNFα agents, including those used for the treatment of IBD, such as infliximab and adalimumab, have also paradoxically been demonstrated to trigger uveitis, which will be further discussed below [8,13].

### 4.2. Epidemiology

Uveitis is a common ocular EIM, with an incidence of 0.5–3.5% in IBD patients [12]. The typical presentation of uveitis in IBD patients is of anterior uveitis, whereas intermediate and posterior uveitis are less associated with IBD [12]. There is a significant overlap between uveitis, arthritis, and ankylosing spondylitis, which are associated with the HLA-B27 allele [41], with 50–80% of acute anterior uveitis patients possessing the HLA-B27 gene [42,43]. Uveitis in IBD patients is also associated with HLA-B58 and HLA-DR103 [8,44].

Uveitis is significantly more common in patients with CD, with a prevalence of 3.27% (95% CI 2.15–4.39%), compared to a prevalence of 1.60% (95% CI 0.93–2.27%) in UC [35]. Uveitis can appear in all age groups, but cases tend to increase with age [37]. Uveitis is the most common EIM in children with IBD and particularly affects male patients, those with colonic involvement, and those with CD [45]. However, children with IBD are unlikely to develop ocular complications from untreated uveitis, which differs from the more aggressive nature of uveitis in children which is associated with juvenile idiopathic arthritis [45].

### 4.3. Presentation

**Figure 3:**
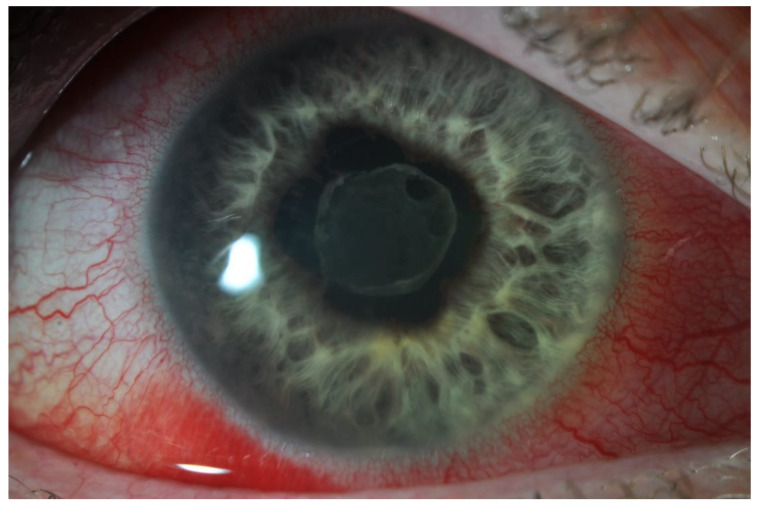
This image demonstrates the irregularly shaped pupil due to posterior synechiae, fibrin formation on the anterior lens surface, and perilimbal flush (redness of the sclera immediately adjacent to the cornea), which is a characteristic of anterior uveitis. The patient will also complain of ocular pain and photophobia. *Image kindly provided by the Manchester Royal Eye Infirmary*.

Patients with anterior uveitis classically present with photophobia, red eye, blurred vision, headache, and ocular pain [39] (Figure 3). Ciliary injection or “perilimbal flush” around the limbus (prominent blood vessels visible around the cornea) is a key feature [1]. Inflammation of the iris may cause adhesions to the lens, known as posterior synechiae, which distort the circular shape of the pupil, leading to irregularity. Patients with IBD tend to present with bilateral uveitic disease (albeit not necessarily simultaneously) and insidious onset of symptoms [8,13].

### 4.4. Management

Patients with suspected uveitis should be referred urgently to the ophthalmology department for management. The inflammation and pain associated with anterior uveitis may be treated with topical corticosteroids and topical cycloplegics, which have the analgesic effect of paralysing the iris and ciliary body as well as reducing the formation of posterior synechiae [8]. It is important to rule out infectious uveitis before commencing steroid treatment [46]. In persistent disease, systemic steroids or immunosuppression are indicated [8].

## 5. Corneal Disease

### 5.1. Overview

Patients with IBD exhibit significant corneal thinning and reduced corneal volume compared to healthy controls [47], lacrimal gland dysfunction [48], and increased risk of corneal inflammation [49]. This combination of factors can lead to dry eye disease, marginal keratitis, and peripheral ulcerative keratitis (PUK) [11]. Recognition of the risk of corneal disease is, therefore, an important part of IBD work up [50].

The pathophysiology leading to corneal EIMs is not fully understood but in general is believed to result from the complex interaction between immune dysregulation, genetic factors, and environmental factors present in IBD [47]. The breakdown of immune tolerance and release of pro-inflammatory cytokines, such as TNF-α and interleukins, can disrupt the normal immune balance of the cornea [51]. Other contributing factors to corneal disease include nutritional deficiencies due to malabsorption. For example, vitamin A deficiency in short bowel syndrome can cause keratoconjunctivitis sicca and keratitis [52,53].

### 5.2. Epidemiology

Corneal disease, or keratopathy, is slightly more prevalent in patients with CD compared to UC, with precise rates varying by pathology [54]. For example, dry eye disease has an incidence rate of 8.18 per 1000 person years in IBD compared to 5.42 per 1000 person years in a healthy population (*p* < 0.01), with more serious corneal damage affecting 2.34 IBD patients per 1000 person years and 2.02 healthy patients per 1000 person years (*p* < 0.01) [48]. Patients are at higher risk of corneal complications during periods of heightened disease activity, as well as in patients with longer disease duration and higher severity. Corneal disease rarely precedes the IBD diagnosis [12]. Patients with extensive colonic disease and those with other EIMs (for example, rheumatological or dermatological) are also at increased risk [8].

### 5.3. Keratoconjunctivitis Sicca

The most common corneal manifestation of IBD is keratoconjunctivitis sicca (KCS), also known as dry eye disease (DED), which leads to dryness of both the cornea and conjunctiva. KCS in IBD patients has an adjusted hazard ratio of 1.43 (95% CI 1.35–1.51) compared to the healthy population and is particularly prevalent in older patients, females, and those with long-standing IBD [48,54]. The use of 5-aminosalicylates is also correlated with increased rates of KCS [13].

The symptoms of irritation, dryness, and foreign body sensation are caused by inflammation of the lacrimal glands and reduced tear production. Mild cases can be managed with artificial tears, punctal plugs (inserted into the lacrimal duct to reduce tear drainage), or topical immunosuppressive agents, including cyclosporine [55]. Vitamin A replacement, taken either orally or intramuscularly, may be effective in cases of deficiency [56]. Severe cases are an indication that further systemic control of IBD is required. Although often considered a mild disease, KCS can have a debilitating impact on quality of life, physical function, and productivity loss, and should, therefore, prompt consideration of onward referral [48].

### 5.4. Peripheral Ulcerative Keratitis

Peripheral ulcerative keratitis (PUK) is a rare but serious inflammatory condition in which ulceration of the peripheral cornea leads to thinning. Its aetiology can be idiopathic or in association with systemic inflammatory conditions [57]. The corneal periphery is particularly susceptible to ulceration due to its physiology, relating to the presence of collagen bundles and the vasculature [58]. PUK frequently presents with unilateral disease but can affect both eyes [12].

PUK can be suspected in patients presenting with painful vision loss who may also complain of excessive tearing and photophobia. A peripheral corneal ulcer may be seen on slit lamp examination with the use of fluorescein to visualise epithelial defects, as well as inflammatory cells in the anterior chamber. Severe cases associated with IBD have led to corneal perforation [59]. Concurrent scleritis, episcleritis, conjunctivitis, or iritis may also be seen [57].

PUK warrants an urgent referral to the ophthalmology department due to the need to treat it early to reduce the risk of corneal perforation. Management during the acute phase involves systemic immunosuppression using corticosteroids, followed by tapering of steroids to prevent disease recurrence. Topical antibiotics prevent bacterial superinfection, and topical lubrication is also used to minimise stromal loss and maintain ocular surface health [60]. If there are concerns about corneal perforation or worsening ulceration despite maximum medical therapy, early surgical intervention is necessary [57]. Surgical interventions may include a lamellar patch graft, conjunctival resection, or amniotic membrane transplant [60].

## 6. Posterior Segment

While rare, it is also pertinent to note the association of IBD with pathology in the posterior segment of the eye due to its significant risk of irreversible loss of vision [61]. It is well recognised that IBD is linked with an increased risk of thromboembolic vascular events with the deep leg and pulmonary veins the most affected [62,63]. However, albeit rare, this can also occur in both venous and arterial retinal vessels [64,65,66,67]. Alongside this, a further pathology related to the retinal vasculature is retinal vasculitis, which is sporadically presented in literature emphasising its uncommon nature [68].

## 7. Iatrogenic Ocular Disease in IBD

Common anti-inflammatory medications used in IBD can have effects on the eye that the gastroenterologist should be aware of. This section provides an overview of iatrogenic ocular conditions associated with IBD therapies, including steroids, immunomodulators, and biologic agents.

The long-term use of systemic corticosteroids is a well-documented risk factor for the development of cataracts in IBD patients, affecting approximately one-third of patients on long-term corticosteroid treatment [69,70]. Cataracts involve the clouding of the eye’s natural lens, leading to impaired vision. Patients on prolonged corticosteroid therapy may experience gradual vision loss and require regular ophthalmic monitoring to detect early cataract formation. Management typically involves surgical intervention, such as phacoemulsification with intraocular lens implantation, when cataracts significantly impair vision [71].

Long-term corticosteroid use can also lead to glaucoma, a condition characterised by irreversible optic neuropathy due to increased intraocular pressure (IOP). A trial of 50 IBD patients treated with prednisolone or budesonide for >4 weeks found 24% of patients developed ocular hypertension, which is the primary risk factor for glaucoma, compared to 2.7–3.8% of the general population who have ocular hypertension [72]. CD patients are more at risk than those with UC [72]. If left untreated, glaucoma leads to permanent loss of vision; therefore, early detection through IOP monitoring in patients on long-term steroids is important. Treatment of glaucoma aims to reduce disease progression and involves a variety of IOP-lowering medications or surgical interventions, such as trabeculectomy, to relieve the pressure [72].

Indirectly, steroid use, which leads to the dysregulation of glycaemic control, can have ophthalmic effects in diabetic patients through the development of diabetic retinopathy [20]. Retinopathy can lead to symptoms such as blurred vision, floaters, or sudden vision loss in the case of vitreous haemorrhage. Retinal detachment can also occur, leading to permanent loss of vision unless surgical intervention is undertaken. Management is focused on optimising glycaemic control, with further anti-VEGF therapies recommended for advanced disease to prevent neovascularisation of the retina [73].

IBD treatment may include methotrexate, azathioprine, and 6-mercaptopurine. Of these, methotrexate is most frequently associated with ocular side effects. Methotrexate is a dihydrofolate reductase inhibitor and reduces the synthesis of purine. Methotrexate can be passed into the composition of tears and is irritating to the cornea and conjunctiva, causing burning, irritation, and general pruritis [8]. Patients on methotrexate may also be at risk of conjunctivitis and blepharitis (inflammation of the eyelid margin) [74]. Azathioprine and 6-mercaptopurine are purine analogues that interrupt DNA and RNA synthesis. They have few documented ocular adverse effects. Azathioprine is in fact commonly used to treat ocular inflammatory conditions, such as scleritis and uveitis [75]. However, there have been isolated cases of ocular toxoplasmosis reactivation and cytomegalovirus retinitis in patients with immunosuppression due to azathioprine treatment [74,76].

Ozanimod is a sphingosine l-phosphate (S1P) receptor modulator used in UC. In a phase 1 placebo-controlled study [77] and a phase 2 uncontrolled trial ‘STEPSTONE’ [78], evaluating the use of ozanimod in healthy volunteers and CD patients, respectively, there were no significant ophthalmological disturbances. However, there were concerns of macular oedema in further trials, with 2 out of 170 patients in a phase 2 placebo-controlled trial and 3 out of 1026 patients in a phase 3 placebo-controlled trial developing this pathology [79,80]. Reassuringly, macular oedema resolved after treatment discontinuation, and the overall incidence is low [80,81]. Macular oedema manifests as blurred vision and occasionally with the visual distortion of objects to appear smaller than they really are [11]. Given there is an association, albeit rare, between ozanimod and macular oedema, an ophthalmology referral for investigation and treatment alongside consideration of discontinuation of ozanimod would be recommended in potential cases [81]. For this reason, consideration of an ophthalmological review for patients with diabetes, uveitis, or a known history of macular oedema before commencing treatment with ozanimod may be appropriate [81,82].

Vedolizumab, an α4β7 integrin-targeting monoclonal antibody, and ustekinimab, a human IL-12 and IL-23 antagonist, are biologic agents used in IBD. The phase 3 open-label long-term safety study ‘GEMINI’ assessed the risk of vedolizumab in IBD and noted 4.2 (95% CI 1.8–6.6) episodes of uveitis per 1000 person years in UC patients and 3.4 (95% CI 1.5–5.2) in CD [83]. No ocular adverse effects were recorded in the ‘UNITI’ placebo-controlled trial exploring the role of ustekinimab during induction and maintenance therapy for CD [84].

Additionally, in a systematic review, the proportion of patients with IBD taking vedolizumab and ustekinimab who developed new ocular manifestations (episcleritis, scleritis, or uveitis) was similar for each drug. The vedolizumab group had an incidence of 1% (95% CI, 0–2%, I^2^ = 36%, 95% CI 0–71%), and the incidence for ustekinimab was also 1% (95% CI, 0–5%, I^2^ = 61%, 95% CI 0–87%), with no significant difference between the groups (*p* = 0.834) [6,85]. In addition, ustekinimab was also associated with an improvement of pre-existing ocular EIMs in 59% of cases (95% CI 32–81%, I^2^ = 0%, 95% CI 0–85%), and there was no worsening of ocular disease. There is similar improvement seen in patients taking vedolizumab; however, this is limited by a significantly smaller sample size [85].

It is pertinent to note that despite the positives mentioned, the choice of medication may influence the likelihood of a patient developing EIMs in the first place. This is highlighted in a study comparing the use of vedolizumab against anti-TNFα medication, where it was noted that patients with CD treated with vedolizumab were more likely to develop episcleritis or scleritis (incidence rate ratio 2.51, 95% CI, 1.02–6.14) and uveitis (incidence rate ratio, 2.89; 95% CI, 1.35–6.18) when compared with those taking anti-TNFα medication [86]. Though this was similarly seen in UC, the results were not statistically significant [86]. It is notable that vedolizumab is a gut-selective agent without systemic immunomodulatory effects, and, therefore, the significant association between the drug and ocular inflammation is not fully understood. It is suggested that the higher likelihood of uveitis, scleritis and episcleritis in patients treated with vedolizumab is due to either unmasked IBD flares in patients with loss of response to therapy or by the mechanism of action of vedolizumab, in which the binding of leukocyte α4β7 integrin to mucosal addressing cellular adhesion molecule-1 (MAdCAM-1) reduces leukocyte migration to the GI tract. This may redirect α4β7-expressing lymphocytes systemically, thus causing extraintestinal effects [86].

Anti-TNFα medications, commonly used for IBD, are also often used in the treatment of various ophthalmic conditions. Golimumab [87,88,89], adalimumab [90,91], and infliximab [32] have all been utilised in the treatment of scleritis and uveitis. However, ocular complications are among the variety of paradoxical autoimmune reactions that can be induced by anti-TNFα drugs (paradoxical because they mimic the autoimmune conditions they are designed to suppress). Paradoxical autoimmune reactions come in many forms, commonly psoriasiform skin lesions and arthralgia [92], but may less commonly include ocular inflammatory diseases, such as uveitis [93]. Literature reviews of anti-TNFα drugs also reference cases of ocular malignancy, herpes zoster keratosis, scleritis, necrotising fasciitis, retinal vein occlusions, optic neuritis, and de novo or relapse of existing chronic uveitis [93,94]. The majority of these cases were in relation to adalimumab, infliximab, or both. However, one study found optic neuritis occurring in a small proportion of patients treated with golimumab or certolizumab [95]. Further investigation is warranted to determine whether this is a true association, as golimumab and certolizumab were licensed more recently than adalimumab and infliximab, so there are less data available regarding the safety profile [93,96]. Alongside this, many of the cases reported are those in which anti-TNFα medications were used to treat a range of systemic inflammatory conditions, such as rheumatoid arthritis and psoriasis, so it remains difficult to identify the risk for ocular adverse side effects in those with IBD as the main treatment indication.

Upadacitinib is a selective JAK-1 inhibitor used in both UC and CD. Filgotinib, also a selective JAK-1 inhibitor, and tofacitinib, a pan-JAK inhibitor, are utilised in the treatment of UC. There were no reported ocular adverse effects in the randomised controlled trials evaluating the respective medications for induction and maintenance of UC and CD (for upadacitinib) and UC (for filgotinib and tofacitinib) [97,98,99,100].

Interestingly, there have been case studies, albeit rare, of patients developing cytomegalovirus (CMV) retinitis following upadacitinib and tofacitinib treatment [101]. CMV retinitis is an opportunistic infection that is sight threatening and warrants urgent referral to ophthalmology. Furthermore, there was one case of retinal vein occlusion out of 102 patients who reported treatment-emergent adverse effects in ‘FINCH 2′, a phase 3 RCT evaluating the effects of filgotinib in patients with rheumatoid arthritis (RA) [102]. Clinicians may be concerned about the increased risk of venous thromboembolism (VTE) seen in IBD patients [103] and the potential role of JAK inhibitors in increasing said risk from an ocular perspective; however, the evidence to support the association of JAK inhibitors and the increased risk of VTE in this population remains ambiguous [62]. This is reinforced by the lack of ocular VTE events reported in the RCTs specific to IBD patients [97,98,99,100].

It must be highlighted that in all the cases mentioned above, the patients were being treated for RA, and further evaluation into the specific cumulative risks of CMV retinitis and RVO for those who are being treated for IBD would be useful to understand whether there is any difference in the risks to this specific population.

In many of the biologic medications currently used in IBD, there is an increased risk of opportunistic infections [97,98,100], such as CMV, which can present with ocular manifestations [104]. Therefore, any patient presenting with symptoms of reduced visual acuity and visual field loss during treatment should be appropriately assessed and referred for urgent investigation wherever necessary.

## 8. The Role of Ophthalmology

In the Emergency Eye Care Commissioning Guidelines published by the Royal College of Ophthalmologists in 2020, there is acknowledgment of the association scleritis and uveitis have with systemic inflammatory conditions [105]. The recommendation as part of management includes early assessment for potential systemic disorders and collaboration with respective specialists. However, there are no specific guidelines on referral pathways or investigations, which may further prompt clinicians to explore this possibility. The lack of a similar recommendation for episcleritis is likely due to the self-limiting nature of the condition; although, the aforementioned recommendation may be relevant in refractory cases.

This recommendation is supported by a large UK-based cohort analysis, showing that patients diagnosed with ocular conditions, including anterior uveitis, episcleritis, and scleritis, are at a twofold increased risk of subsequent diagnosis of IBD, especially CD [106]. The delay between the diagnosis of the ocular condition to the diagnosis of IBD was, on average, greater than two years [106]. This demonstrates a significant delay in possible diagnosis and treatment of IBD. The non-specific correlation of symptoms highlights the importance of a thorough history and systems review in both the ophthalmology setting and in general practice. Further simple investigations, such as faecal calprotectin, and onward gastroenterology referral should be considered to reduce the risk of delayed diagnosis and treatment, especially in those presenting with gastrointestinal symptoms, including rectal bleeding, anaemia, and loose stool, which were common in patients presenting for the first time with ocular EIMs but without a formal IBD diagnosis [106].

## 9. Conclusions

In summary, understanding and addressing ocular EIMs is important to help aid early diagnosis and improve comprehensive IBD management (see Table 1). Advanced therapies used to treat IBD carry an increased risk of developing ocular complications, ranging from the common conditions, such as cataracts and glaucoma, to the less common but severe manifestations, such as optic neuritis.

There are aspects of this topic that require further research. It is suggested that future research may focus on addressing the precise pathophysiological mechanisms of ocular EIMs, in particular scleritis and corneal disease. Broader research into the risks of new IBD medications, such as ozanimod, vedolizumab, and JAK inhibitors, is also a priority. This may focus on establishing the underlying mechanism of ocular side effects and differentiating risks that are specific to IBD patients from those that have been found for the same drugs used in other autoimmune conditions. For example, is there a significant association between the anti-TNFα agents golimumab and certolizumab and the development of optic neuritis? And, do JAK inhibitors increase the risk of retinal vein occlusion or CMV retinitis?

The development of protocols for both ophthalmological monitoring of high-risk IBD patients and GI investigations for patients with recurrent ocular activity suspicious of underlying systemic disease would also be welcome. Ongoing surveillance and vigilant assessment will be essential as newer therapies become more widely used, especially in patients with pre-existing risk factors for ocular disease. A thorough history and systems review will facilitate the early detection of patients who will benefit from an ophthalmology referral, thus improving holistic management and enhancing patient outcomes. Indeed, as the prevalence of IBD continues to rise, collaboration between the gastroenterology and ophthalmology teams is imperative to allow earlier recognition and treatment of these conditions.

## Figures and Tables

**Table 1:** A summary of ocular extraintestinal manifestations of inflammatory bowel disease, including relevant epidemiological and clinical information for the gastroenterologist.

EIM	Incidence in IBD	Risk Factors	Presentation	Management
Episcleritis	2–5% CD > UC in European populations CD = UC globally	Active IBD flares Other autoimmune diseases	Mild–moderate ocular discomfort Episcleral hyperaemia, itching	Conservative management + treatment of underlying disease Cool compress, topical lubrication Topical NSAID, topical corticosteroids Oral NSAIDs
Scleritis	<1% CD > UC	Other autoimmune diseases Long-term corticosteroids Increased age Female Black/South Asian	Moderate–severe ocular pain Scleral hyperaemia Blurred vision Ocular tenderness	Oral NSAIDs Oral corticosteroids Systemic immunosuppressants, e.g., methotrexate +/− topical corticosteroid
Uveitis	0.5–3.5% CD > UC	Ankylosing spondylitis HLA-B27, HLA-B58, HLA-DR103 Increased age Male Colonic involvement Anti-TNFα agents Not associated with active IBD flares	Photophobia Red eye Blurred vision Headache Eye pain Conjunctival injection	Topical steroids Topical cycloplegics Systemic steroids and immunosuppression if severe
KCS	Up to 44%	Increased age Females Long-standing IBD Use of 5-ASA	Irritation Dryness Foreign body sensation	Artificial tears Punctal plugs Topical immunosuppressants Vit A replacement
PUK	Rare	Increased age Systemic autoimmune disease	Ocular pain Vision loss Ulceration seen on slit lamp examination with fluorescein	Corticosteroids Topical antibiotics Lubrication
